# A Modified Thyroid Imaging Reporting and Data System (mTI-RADS) For Thyroid Nodules in Coexisting Hashimoto’s Thyroiditis

**DOI:** 10.1038/srep26410

**Published:** 2016-05-19

**Authors:** Hang Zhou, Wen-Wen Yue, Lin-Yao Du, Jun-Mei Xu, Bo-Ji Liu, Xiao-Long Li, Dan Wang, Xian-Li Zhou, Hui-Xiong Xu

**Affiliations:** 1 Department of Medical Ultrasound, Shanghai Tenth People’s Hospital, Ultrasound Research and Education Institute, Tongji University School of Medicine, Shanghai 200072, China; 2 In-Patient Ultrasound Department, The Second Affiliated Hospital of Harbin Medical University, Harbin 150086, China; 3 Thyroid Institute, Tongji University School of Medicine, Shanghai 200072, China

## Abstract

To develop a conventional ultrasound (US) modified Thyroid Imaging Reporting and Data System (mTI-RADS) to stratify the malignancy risk of thyroid nodule in coexisting Hashimoto’s thyroiditis (HT). The study included 138 malignant and 292 benign thyroid nodules confirmed by cytological or histopathological results. The risk score (RS) for each significant US feature was estimated by multiplying corresponding regression coefficient and the total score for each nodule was defined as the sum of these individual scores. The mTI-RADS was established according to the total RS and divided into category 3, 4a, 4b, 4c and 5. Marked hypoechogenicity, taller-than-wide shape, poorly-defined margin, microcalcification or macrocalcification and halo sign absence were statistically significant US features in prediction of thyroid malignancy (all *p* < 0.05). The total RS for each nodule was defined as following: RS = 2.1× (if marked hypoechogenicity) + 1.2× (if taller-than-wide shape) + 1.7× (if no halo sign) + 0.6× (if poorly-defined margin) + 1.2× (if microcalcification or macrocalcification). The malignancy rates in mTI-RADS category 3, 4a, 4b, 4c and 5 nodules were 3.7%, 19.3%, 38.1%, 62.7% and 94.1%, respectively, with significant differences among different categories (*P* < 0.001). The mTI-RADS category may facilitate subsequent treatment management in HT patients.

Hashimoto’s thyroiditis (HT) is a chronic autoimmune inflammation disease of thyroid gland which is characterized by lymphocyte infiltration and fibrosis. Compared with non-HT patients, HT patients have been proven to be more positively associated with thyroid malignancy^1^,^2^,^3^,^4^,^5^. Larson *et al.* stated that increasing PI3k/Akt expression in both HT and well-differentiated thyroid cancer (TC) might be a possible molecular mechanism for thyroid carcinogenesis^6^. Ultrasound (US) is the first-line imaging modality for screening thyroid nodules coexistent with HT due to its high resolution, convenience, no radiation, wide availability, and cost-effectiveness. On US, the thyroid gland is highly suggestive of HT when diffuse, coarse or heterogeneous background, micronodulation, echogenic separations and decreased echogenicity are found^7^,^8^,^9^,^10^,^11^,^12^.

US-guided fine needle aspiration (FNA) is an efficient method for evaluating thyroid nodules, which greatly avoids unnecessary surgical interventions for benign nodules. Solid component, internal calcifications, marked hypoechogenicity, irregular margin, and taller-than-wide shape on US are usually regarded as risk factors for thyroid malignancy^13^,^14^,^15^,^16^. The American Thyroid Association (ATA) and American Association of Clinical Endocrinologists, Associazione Medici Endocrinologi and European Thyroid Association (AACE/AME/ETA) have issued several medical guidelines on how to select suspicious thyroid nodules on US for further FNA investigation^17^,^18^. On the other hand, several US Thyroid Imaging Reporting and Data Systems (TI-RADSs) have been proposed for risk stratification of thyroid nodules. The nodules are usually divided into different categories based on the TI-RADS and then referred to FNA or follow-up according to different malignancy risks^19^,^20^,^21^,^22^,^23^. The concept of TI-RADS mimicks that of Breast Imaging Reporting and Data System (BI-RADS)^24^. However, until present, no thyroid guidelines or TI-RADS categorizations have focused on management of nodules coexistent with HT which might predispose to TC more likely.

Therefore, this retrospective study was to identify the risk US features associated with TCs in HT patients and to establish a modified TI-RADS (mTI-RADS) based on conventional US that could be applied for nodules coexistent with HT.

## Materials and Methods

This retrospective study was approved by the Ethics Committee of Shanghai Tenth People’s Hospital and the requirement to obtain informed consent from each patient for data analysis was waived . The study was performed in accordance with Declaration of Helsinki for human study.

### Patients

From October 2011 to March 2015, a consecutive of 6958 patients with nodule size ≥5 mm underwent thyroid US examination and FNA and/or surgery in this referral hospital. The patient exclusion criteria were as follows: (a) with previous history of invasive procedures on thyroid (n = 166); (b) non-HT patients (n = 6228); (c) HT patients with less than 6 months’ follow-up after obtaining benign results on FNA cytology (n = 58); (d) HT patients without final histopathological results if FNA cytological results were classified as nondiagnostic (ND), atypia of undetermined significance (AUS) or follicular lesion of undetermined significance (FLUS), follicular neoplasm (FN) or suspicious for follicular neoplasm (SFN), suspicious for malignancy or malignancy (n = 100); (e) HT patients with entirely calcified thyroid nodules in which the US features could not be analyzed properly due to posterior acoustic shadowing (n = 5). Finally, the study group consisted of 307 pathologically proven nodules and 123 cytologically proven nodules in 401 HT patients (female-to-male ratio: 10.14). They were 376 patients with solitary nodule, 21 patients with two nodules and 4 patients with three nodules.

### US examination

Conventional US was performed with Siemens S2000 (Siemens Medical Solutions, Mountain View, CA, USA; 5–14 MHz linear probe), IU22 (Philips Medical Systems, Bothell, WA, USA; 5–12 MHz linear probe) or Logiq E9 (GE Medical Systems, Milwaukee, WI, USA; 6–15 MHz linear probe) instruments by three radiologists who were board certified with at least 4 years’ experience in thyroid US. All the US examinations were complied with the same protocol for thyroid scanning. The patient lied on the bed in supine position with slight dorsal flexion of the head. Conventional US images of the thyroid nodule were acquired by carefully scanning the thyroid and adjacent tissues both transversely and longitudinally. The nodule’s size was defined by the maximal diameter at US. The radiologist selected the suspicious nodules (if any one of the features such as hypoechogenicity, microcalcification, irregular margin, intranodular vascularity, taller-than-wide shape) for evaluation^18^. If multiple nodules were present, the most suspicious ones would be targeted. For multiple nodules without suspicious nature, the largest one would be evaluated. The machine settings were optimized to obtain US images that showed the optimal imaging features and then the images were stored in the internal hard-disk of the instruments for further analysis.

### Retrospective Interpretation

Three radiologists reviewed all the US images independently, who had 2, 5, and 6 years of experience, respectively, in thyroid US. None of them was involved in image acquisition of the study cohort. A training session was carried out before formal interpretation. All reviewers were asked to assess the US characteristics when evaluating 30 pathologically-confirmed thyroid nodules in 30 HT patients which were excluded from final study population. Then the three radiologists discussed a baseline consensus in lexicon for US characteristics. The US characteristics included internal component, nodule echogenicity, calcification, shape, margin, and halo sign. The internal component was categorized according to the ratio of the cystic portion to the solid portion in the nodule as complete solid, predominantly solid (≤50% cystic portion) and predominantly cystic (>50% cystic portion)^20^. The echogenicity was interpreted according to the solid portion in the nodule and was compared with adjacent thyroid parenchyma or strap muscle. The echogenicity was defined as hyperechogenicity (higher echogenicity than adjacent thyroid parenchyma), isoechogenicity (equal echogenicity with adjacent thyroid parenchyma), hypoechogenecity (lower echogenicity than adjacent thyroid parenchyma) and marked hypoechogenicity (lower echogenicity than the adjacent strap muscle)^16^,^21^,^25^. The calcifications were classified as microcalcifications (tiny, punctuate echogenic foci of 1 mm or less either with or without posterior shadowing), macrocalcifications (punctuate echogenic foci larger than 1 mm in size), and no calcification. The rim calcification around the nodule was deemed to be macrocalcification. When micro- and macro-calcifications were observed in the same nodule, we regarded it as microcalcifications^16^,^21^,^22^. The nodule shape was defined as taller-than-wide (greater in its anteroposterior dimension than in its transverse dimension) or wider-than-tall^21^,^26^. The margin of the nodule was categorized as well-defined when clear demarcation was noted around more than 50% of a nodule or poorly-defined when more than 50% of the border of the lesion was not clearly demarcated^27^. The halo sign was defined as a hypoechoic rim around a nodule^20^.

Then all reviewers individually performed retrospective analysis of US images in the formal study session without knowledge of others’ results. Patients’ medical information (previous imaging results and cytological or histopathological results) were blinded to the three reviewers either in the training session or formal session. Disagreement was resolved by final consensus.

### Reference standard

The diagnosis of HT was confirmed by heterogeneous echogenicity of thyroid gland on US in combination with cytological and/or histological results. Echogenicity was considered to be heterogeneous when the thyroid parenchyma exhibited one or more of the following features: diffuse, coarse or heterogeneous echogenicity, micronodules, linear echogenic separations^8^,^11^,^12^,^28^,^29^,^30^. US-guided FNA was performed with a 23-gauge PTC needle attached to a 5-mL disposable plastic syringe. Each lesion was aspirated at least twice. Materials obtained from aspiration biopsy were expelled onto glass slides and were then smeared. All smears were placed immediately in 95% alcohol for hematoxylin-eosin staining. Cytopathologists were not on site during the biopsy. The cytological results of the thyroid nodules were in coordination with Bethesda system as ND, benign (including HT nodule), AUS or FLUS, FN or SFN, suspicious for malignancy or malignancy^31^. HT was diagnosed on FNA when the cytological specimen met the following criteria: grouped, monolayer sheets or scattered follicular and Hurthle cells with scattered lymphocytes; the colloid was scanty; and the follicular cells showed nuclear atypia with nuclear enlargement and clearing in the absence of nuclear grooves or inclusions^32^.

The indications referred for surgical intervention were as follows: nondiagnostic (n = 36), AUS (n = 26), FN or SFN (n = 15), suspicious for malignancy (n = 42), malignant (n = 18) on FNA results, patient anxiety (n = 80), compressive symptoms or discomfort caused by the large nodules or the associated large nodules (n = 90).

The clinical outcome (benign or malignant) of nodule in HT patient was determined by FNA or surgical specimen. When both cytological and histopathological results were acquired in the same patient, the latter one was considered as the reference standard. The duration of imaging follow-up with US for the nodules with initially benign FNA results was at least 6 months (range: 6–24 months) and nodule stability (no more than a 50% change in volume or <20% increase in at least two nodule dimensions in solid nodules or in the solid portion of mixed cystic–solid nodules) was confirmed as American Thyroid Association guidelines demonstrated^31^.

### Statistical Analysis

Statistical analyses were performed with SPSS software for Windows (version19.0; Chicago, IL, USA). Patient age and nodule size were compared by independent *t* test. Categorical variables were compared by Chi-square test or Fisher exact probability test if necessary, including each US feature and patient sex. In addition, US predictors for malignancy that showed statistical significance were determined by the method of multiple logistic regression analysis with a forward stepwise selection. Odds ratios (ORs) with relative 95% confidence intervals (CIs) were also calculated to determine the relevance of all potential predictors for malignancy. The risk score (RS) for each significant US feature was multiplied by the regression coefficient (β) obtained from multivariate logistic regression analysis and the score of malignancy for each nodule was defined as the sum of these individual scores. All regression coefficients were standardized to make the scores approach one decimal place. The mTI-RADS was determined from the lowest to the highest total RS into five categories (category 3, category 4a, category 4b, category 4c, and category 5). For statistical analysis, mTI-RADS category 3 nodules were considered as benign and mTI-RADS category 4 or 5 as malignant. The diagnostic performance of the mTI-RADS category (sensitivity, specificity, positive and negative predictive value) was calculated. The Spearman rank test was used to evaluate the relationship between each category and predicted probability for TC obtained from the regression analysis. Statistical significance was determined at a *P* value less than 0.05.

Inter-observer agreement was assessed for each US feature using the guideline of Landis and Koch for interpreting K values: slight agreement (0.00–0.20), fair agreement (0.21–0.40), moderate agreement (0.41–0.60), substantial agreement (0.61–0.80), and almost perfect agreement (0.80–1.00)^33^.

## Results

### Pathological and cytological results

All malignant nodules included in this study were confirmed by histopathological diagnosis (n = 138). Of them, 136 (98.6%) were papillary thyroid carcinomas (PTCs) and 2 (1.4%) were medullary carcinomas. There were 123 benign nodules confirmed by cytopathology in combination with follow-up and 169 benign nodules confirmed by histopathology, including Hashimoto nodules (n = 87), nodular goiters (n = 55) and adenomas (n = 27) in HT patients.

### Basic demographic characteristics in predicting TC

The basic characteristics of the patients and the nodules are shown in Table 1. Malignant nodules were significantly smaller than benign ones (*p* < 0.001). Thyroid malignancy was more commonly found in younger patients (*p* < 0.001). On the other hand, gender and nodule location were not associated with TC in HT patients (both *p* > 0.05).

### Conventional US features in predicting TC

The US features for the benign and malignant nodules are illustrated in Table 1. On multivariate logistic regression analysis, the following US features were identified to be significant predictors for TC: marked hypoechogenicity (OR = 7.783; 95% CI: 1.413–42.875), taller-than-wide shape (OR = 3.364; 95% CI: 1.963–5.764), poorly-defined margin (OR = 1.775; 95% CI: 1.069–2.947), microcalcification (OR = 3.418; 95% CI: 2.080–5.617) or macrocalcification (OR = 3.377; 95% CI: 1.040–10.964), and absence of halo sign (OR = 5.631; 95% CI: 1.187–26.705)(all *p* < 0.05). Among them, marked hypoechogenicity was the most significant predictors. The regression coefficients of the five significantly suspicious US features are shown in Table 2.

### mTI-RADS category in risk-stratification for thyroid nodules

**Figure 1:**
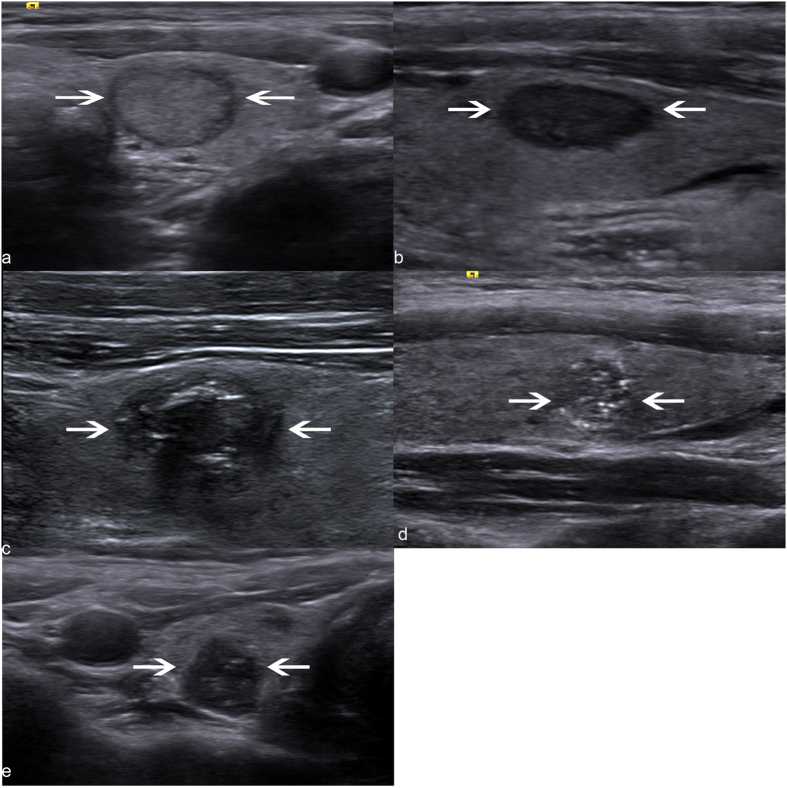
US-based mTI-RADS categories in HT patients. (**a**) Category 3. US image of a cytologically benign nodule (arrows) shows hypoechoic rim around the nodule without any suspicious US features. (**b**) Category 4a. US image of a cytologically benign nodule (arrows) shows a hypoechoic lesion (arrows) without halo sign. (**c**) Category 4b. US image shows a poorly-defined and hypoechoic nodule (arrows) with microcalcifications and absence of halo sign, which is finally proven to be a papillary thyroid carcinoma pathologically. (**d**) Category 4c. US image shows a poorly-defined, taller-than-wide and hypoechoic nodule with microcalcifications whereas without halo sign, which is finally proven to be a papillary thyroid carcinoma pathologically. (**e**) Category 5. US image shows a taller-than-wide, marked hypoechoic nodule (arrows) with microcalcification whereas without halo sign, which is finally proven to be a papillary thyroid carcinoma pathologically.

**Figure 2:**
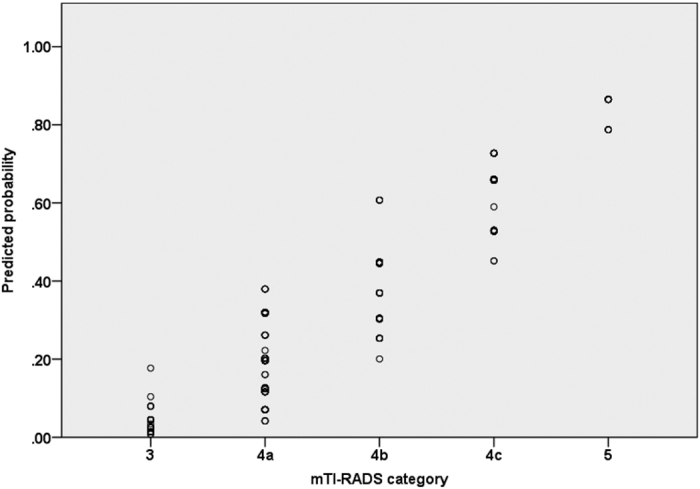
Scatter plots of predicted probability for thyroid malignancy by mTI-RADS category. X-axis: mTI-RADS category; Y-axis: the predicted probability of thyroid cancer calculated from multivariate regression analysis.

A final predicting model was established on the basis of the five risk factors derived from multivariate logistic regression analysis. The sum of RS for each nodule was defined as follows: RS = 2.1× (if marked hypoechogenicity) + 1.2× (if taller-than-wide shape) + 1.7× (if absence of halo sign) + 0.6× (if poorly-defined margin) + 1.2× (if microcalcification or macrocalcification) (Table 2). The total RS for each nodule ranged from 0 to 6.8. Then the mTI-RADS scoring system was divided into 5 categories according to the total RS: mTI-RADS 3 (very low risk, 0 ≤RS < 1.5), 4a (low risk, 1.5 ≤RS < 3), 4b (moderate risk, 3 ≤RS < 4.5), 4c (high risk, 4.5 ≤RS < 6) and 5 (very high risk, RS ≥ 6) (Fig. 1). The malignancy rates were 3.7%, 19.3%, 38.1%, 62.7%, and 94.1% for category 3, 4a, 4b, 4c, and 5, respectively, with significant differences among different categories (all *p* < 0.001)(Table 3). A linear relationship between mTI-RADS category and the predicted probability of TC was established (Fig. 2). The predicted probability of thyroid malignancy increased with elevated mTI-RADS category with an *r* value of 0.926 (*p* < 0.001). The sensitivity, specificity, positive and negative predictive values of mTI-RADS were 98.6% (136/138), 17.8% (52/292), 36.2% (136/376), 96.3% (52/54), respectively, when considering category 3 as negative and categories 4a to 5 as positive results.

### Reviewer Agreement in interpreting US features

Between senior reviewers (Reader 2 and 3), a substantial agreement was obtained for interpreting shape or halo sign whereas a moderate agreement for component, echogenicity, calcification and margin. For the junior and senior reviewers (Reader 1 vs. Reader 2 or 3), a substantial agreement was reported for shape and halo sign, whereas moderate agreement for echogenicity and component, a substantial or higher agreement for calcification and a fair to moderate agreement for margin (Table 4).

## Discussion

Increasing detection of thyroid nodules through US examination justifies the need of US-based risk stratification system to determine subsequent management strategy such as FNA or follow-up. It has been reported that there may be some differences in US features between thyroid nodules with HT and those without HT. Pathologically, an increased incidence of dense calcifications and a decreased incidence of psammoma bodies in TC were found to be associated with HT patients compared to those without^34^. With regard to US manifestations, Park *et al.*^35^ found that microlobulated or irregular margins of benign thyroid nodules were more frequently seen in thyroid glands with heterogeneous echogenicity background that was often encountered in HT patients, in comparison with those with homogenous echogenicity background. Durfee *et al.*^36^ also found that among patients with HT and TC, although the US appearance of the cancerous nodule was similar, the cancerous nodule margins were more likely to be irregular or poorly defined when the gland was heterogeneous. Therefore, it is necessary to develop an US-based risk stratification system dedicated to HT patients in consideration of the elevated malignant risk and possible different US patterns in those patients.

The present study suggested that marked hypoechogenicity, taller-than-wide shape, poorly-defined margin, microcalcification or macrocalcification and absence of halo sign were independent US features in prediction of thyroid malignancy with HT, which were consistent with other published literatures^15^,^20^. However, some authors found that solid component significantly increased the likelihood of malignancy in general population^13^,^21^ whereas it was not found in HT patients. In our study, most benign nodules (82.9%, 242/292) exhibited solid appearance as well as malignant ones (94.9%, 131/138). We hypothesized that the solid component of some benign nodules might be derived from dense fibrosis keloid-like bands which subvert the normal thyroid architecture and impart to the gland a lobular appearance^10^. Hence, a solid appearance alone was failed to be identified as a predictor for TC in HT patient, which indicates that a dedicated mTI-RADS for HT patients is necessary from another perspective.

Recently, several TI-RADS classifications have been developed to facilitate communication between clinicians and radiologists, which allow the clinicians to readily understand the malignancy risk of a thyroid nodule in an US report and provide clear guideline for subsequent management (follow-up or biopsy)^19^,^20^,^21^,^22^,^23^, just as BI-RADS did for breast lesions. However, none of them addressed the strategy of choosing which nodule for FNA in patients coexistent with HT. In addition, different methodologies adopted by these categorizations confused the radiologists for their application in clinical practice, such as 10 stereotypic patterns^23^, complex equation of 12 parameters^20^ and same weight risk on different US features^21^. To overcome these methodological drawbacks, Kwak *et al.*^22^ proposed to assign individual risk score (Exp[β]) on suspicious US parameters to create a risk-stratifying model for thyroid nodules, which showed good diagnostic performance in predicting TC with an area under the receiver operating characteristic (ROC) curve of 0.867 (95% CI, 0.846–0.887). Therefore, we conducted this study to establish an mTI-RADS categorization for thyroid nodules coexistent with HT by calculating the RS (β coefficient) for each nodule.

In the present study, we classified thyroid nodules into five categories according to the total RS. The risk of malignancy was 3.7% among category 3 nodules in this study, which was lower than the result of Horvath *et al.* (14.1%)^20^,^23^, whereas higher than that of Kwak *et al.* (1.5%)^21^. The present study also showed a tendency for an increased malignancy rate that was associated with elevated mTI-RADS categorization, as seen in a previous study in which the risk of malignancy increased proportionally to the number of suspicious malignant US features^21^. According to our definition of mTI-RADS, nodules classified as category 3 (very low risk) would be referred to regular US observation or FNA biopsy only when clinically warranted, such as rapidly growth of target nodule, unavailability for follow-up regular checkups or previous history of radiation on neck. On the other hand, patients with mTI-RADS category 4 and 5 nodules should be recommended for FNA biopsy. The sensitivity, specificity, positive and negative predictive values of the mTI-RADS were 98.6% (136/138), 17.8% (52/292), 36.2% (136/376), and 96.3% (52/54), respectively, by dividing the nodules into benign (category 3) and malignant groups (category 4a to 5).

In spite of a prior training process for three reviewers, the inter-observer agreement was only fair to moderate for margin and moderate for echogenicity. The off-site reading of the US images rather than on-site reading might confound one’s judgment of the US features in each nodule. The heterogeneous echogenicity of thyroid parenchyma may be another possible factor affecting the radiologist’s assessment, especially on margin delineation^35^.

Our study had several limitations. Firstly, this single-center study would inevitably lead to selection bias, which could be overcome by multi-center study on HT patients in the future. Secondly, in clinical field, the diagnosis of HT is mainly based upon elevated serum levels of antibodies to thyroglobulin or thyroperoxidase^10^, whereas different diagnostic criteria for HT including both US features and cytological or histopathological examinations were adopted in this study. We deemed it as a minor problem since the purpose of this study was primarily emphasized on how to assess nodules coexistent with HT on abnormal US background, which was neglected by previous studies. For the HT patients with homogeneous echogenicity background on US , conventional TI-RADS may be applicable. Thirdly, due to the retrospective nature of this study, variability in US machines and operators might limit the image interpretation by reviewers. However, all the machines used in this study were high-end instruments and were performed by experienced radiologists. In addition, the thyroid US images were scanned and stored under the same protocol. Thus the influence due to the above-mentioned factors was reduced to a minimal extent whereas a prospective study design is still necessary. Fourth, primary thyroid lymphoma was reported to be associated with HT at a high risk level^37^, which might share different US features from PTC that accounted for 99% of TC in our study population. However, in this study, no primary thyroid lymphoma was encountered. Future studies consisting of various types of thyroid malignancies in HT patients are mandatory in the future. Fifth, we removed the thyroid nodules that US features could not be analyzed properly, such as nodules with entire calcification in which posterior acoustic shadowing would affect the result of image interpretation. Thus the mTI-RADS may not be suitable for such nodules. Finally, to test a probability system, it would be better to have a training set to create a TI-RADS category, then another validation set to test this category^22^, which was absent in this study. A validation study is already started in the center whereas the cases are still limited.

In conclusion, our findings suggest that some US features are helpful for differentiating benign from malignant nodules in patients coexistent with HT, including marked hypoechogenicity, taller-than-wide shape, poorly-defined margin, microcalcification or macrocalcification and absence of halo sign. The established mTI-RADS category has a high sensitivity and may be useful for decision-making with respect to management of thyroid nodules in HT patients. However, a prospective study is needed in the future to validate the effectiveness of this system.

## Additional Information

**How to cite this article**: Zhou, H. *et al.* A Modified Thyroid Imaging Reporting and Data System (mTI-RADS) For Thyroid Nodules in Coexisting Hashimoto’s Thyroiditis. *Sci. Rep.* **6**, 26410; doi: 10.1038/srep26410 (2016).

## Figures and Tables

**Table 1:** Basic demographic characteristics and conventional US features in predicting thyroid malignancy coexistent with HT.

Parameter	Benign (n = 292)	Malignant (n = 138)	P value
Patient			
Gender			0.902
Female	247 (91.1)	118(90.7)	
Male	24 (8.9)	12 (9.3)	
	53.6 ± 11.6 (23–80)	47.0 ± 12.9 (17–77)	<0.001
Nodule			
Mean size(mm)#	14.3 ± 7.8 (5.0–38.0)	10.9 ± 5.3 (5.0–34.4)	<0.001
Location			0.548
Left	128 (44.0)	53 (38.4)	
Right	149 (4.8%)	78 (56.5)	
Isthmus	14 (51.2%)	7 (5.1)	
Component			0.003
Predominantly cystic	6 (2.1)	1(0.7)	
Predominantly solid	44(15.1)	6(4.3)	
Solid	242(82.9%)	131(94.9%)	
Echogenicity			<0.001
Hyperechogenicity	14(4.8)	2 (1.4)	
Isoechogenicity	87 (29.8)	10 (1.2)	
Hypoechogenicity	156 (53.4)	76 (55.1)	
Marked hypoechogenicity	35 (12.0)	50 (36.2)	
Shape			<0.001
Taller than wide	50 (17.1)	65 (47.1)	
Wider than tall	242 (82.9)	73 (52.9)	
Calcification			<0.001
No calcification	198 (67.8)	52 (37.7)	
Macrocalcification	11 (3.8)	5 (3.6)	
Microcalcification	83 (28.4)	81 (58.7)	
Margin			<0.001
Well-defined	180 (61.6)	41 (29.7)	
Poorly-defined	112 (38.4)	97 (70.3)	
Halo sign			<0.001
Present	55 (18.8)	2 (1.4)	
Absent	237 (81.2)	136(98.6)	

HT, Hashimoto’s thyroiditis.

Note - numbers in parentheses are percentages, otherwise are ranges.

^#^Data are means ± standard deviations.

**Table 2:** Risk score assignment of independent US features in predicting thyroid malignancy coexistent with Hashimoto’s thyroiditis on multivariate logistic regression.

Parameter	β	SE	OR (95% CI)	*P* Value	RS	Assignment	
						No	Yes
Marked Hypoechogenicity	2.052	0.871	7.783 (1.413, 42.875)	0.018	2.1	0	1
Microcalcification	1.229	0.253	3.418 (2.080, 5.617)	<0.001	1.2	0	1
Macrocalcification	1.217	0.601	3.377 (1.040, 10.964)	0.043	1.2	0	1
Taller-than-wide Shape	1.213	0.275	3.364 (1.963. 5.764)	<0.001	1.2	0	1
Poorly-defined Margin	0.574	0.259	1.775 (1.069, 2.947)	0.027	0.6	0	1
Absence of Halo Sign	1.728	0.794	5.631 (1.187, 26.705)	0.030	1.7	0	1

Note - β, regression coefficient; SE, standard error; OR, odds ratio; CI, confidence interval; RS, risk score.

**Table 3:** Malignancy rates according to mTI-RADS category in patients coexistent with Hashimoto’s thyroiditis.

mTI-RADS category	Risk score (RS)	No. of thyroid malignancy	No. of total nodules	Malignancy rate (%)
3 (very low risk)	0 ≤ RS < 1.5	2	54	3.7
4a (low risk)	1.5 ≤ RS < 3	36	187	19.3
4b (moderate risk)	3 ≤ RS < 4.5	37	97	38.1
4c (high risk)	4.5 ≤ RS < 6	47	75	62.7
5 (very high risk)	6 ≤ RS	16	17	94.1

Note – mTI-RADS, modified thyroid imaging reporting and data system.

**Table 4:** Inter-observer agreement on the interpretation of US features.

US features	K value		
	Reader 1 vs. Reader 2	Reader 1 vs. Reader 3	Reader 2 vs. Reader 3
Component	0.591	0.563	0.596
Echogenicity	0.427	0.438	0.499
Calcification	0.604	0.586	0.596
Shape	0.696	0.720	0.748
Margin	0.367	0.403	0.510
Halo sign	0.616	0.653	0.638

Note —Reader 1, 2, 3 are reviewers with experience of 2, 5, 6 years, respectively, in thyroid US.
